# A new species of the genus *Teloganopsis* with setaceous mouthparts and forelegs from southern China (Ephemeroptera, Ephemerellidae)

**DOI:** 10.3897/zookeys.714.13646

**Published:** 2017-11-06

**Authors:** Wei Zhang, Zhen-Xing Ma, Ze Hu, Juan-Yan Luo, Chang-Fa Zhou

**Affiliations:** 1 The Key Laboratory of Jiangsu Biodiversity and Biotechnology, College of Life Sciences, Nanjing Normal University, Nanjing 210046, China

**Keywords:** evolution, filter, Mayfly, new species, taxonomy

## Abstract

The nymph and reared male and female of a new ephemerellid species, which was collected from southern China and named *Teloganopsis setosa* Zhou, **sp. n.**, are described. The nymph is unique because of its long and dense setae on labrum, mandibles, maxillae, labium, and forelegs as well as the elongated segments II of labial palpi and expanded paraglossae. The male can be differentiated from close relatives by its larger penis lobes with dorsolateral projections, and the more pigmented tergum IV and caudal filaments. The nymph described in this paper represents a new adaptive and ecological type in the family Ephemerellidae.

## Introduction

Jacobous and McCafferty (2008) redefined the genus *Teloganopsis* Ulmer (1939) and synonymized it with *Amurella* Kluge (1997) (nymphs have single abdominal tubercles), *Uracanthella* Belov (1979) (with brush-like maxillae but without maxillary palpi), and *Kangella* Sartori (2004) (= *Eburella* Kang & Yang, 1995, with enlarged mandible incisors). Subsequently, Ubero-Pascal and Sartori (2009) revised this genus and placed 15 species into it. The nymphs of this genus include several ecological and adaptive types, each with differing morphologies, especially mouthparts. They may be filter-feeders using maxillae (like *Uracanthella*), biters or shredders (*Amurella*), or cutters and predators having enlarged mandibles (*Kangella*).

In this study, a new ecological type and evolutionary lineage of this genus is found in Hainan Island, southern China. The nymphs have large labia, elongated labial palpi with long setae, and forelegs with rows of long setae. The setaceous mouthparts and forelegs show this species can filter particle food items in running water.

## Materials and methods

The nymphs were collected by hand net. Some adults were reared from mature nymphs indoor but most were attracted by ultraviolet collecting light. The materials were stored into ethanol (more than 75%) immediately. All specimens were photographed with a digital camera (Single Lens Reflex) and examined under a stereomicroscope. Some small structures, such as mouthparts, claws, and gills were observed and photographed with a microscope camera.

Eggs were dissected out from females. Before being placed on the stage of the SEM (scanning electron microscope) for photographs, they were prepared with a standard protocol: fixed in 4% glutaraldehyde for 4–8 hours, rinsed with PBS (physiological saline) 2–3 times (10–15 minutes each), dehydrated in concentration gradient acetone (30%, 50%, 70%, 80%, 90%, 100%, 10 to 15 minutes each), and coated with gold film in a vacuum.

Comparative material used in this study includes:

*Teloganopsis punctisetae* (Matsumura, 1931) (=*Ephemerella rufa* Imanishi, 1937, synonymized by Ishiwata, 2001): 2♂♂3♀♀4L, Xin-Huang county (27°19.10′N, 109°14.05′E, alt. 352 m), Hunan Province, China, collected by Peng LI, Jia-Yong ZHANG, 16 Aug 2004.

*Teloganopsis jinghongensis* (Xu et al., 1984): 2♂♂3♀♀3L, Er-Yuan town (26°15.26′N, 99°58.55′E, alt. 2093 m), Da-Li, Yunnan Province, China, collected by Hui XIE, Ping CHEN, Yan-Yan JIA, 7 July 2008.

Abbreviations used in text: **C**, costal vein; **Sc**, subcostal vein; **SEM**, scanning electron microscope.

All specimens are deposited in the Mayfly Collection, College of Life Sciences, Nanjing Normal University, China.

## Results

### Teloganopsis setosa

Taxon classificationAnimaliaORDOFAMILIA

Zhou sp. n.

http://zoobank.org/507C1DC5-0D06-41BF-9320-74B75695901D

**Figure 1.**
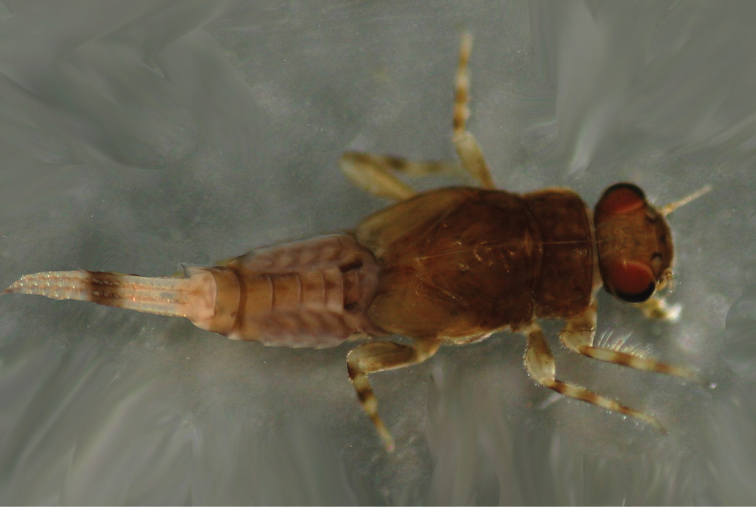
Digital photograph of male nymph of *Teloganopsis setosa* sp. n.

**Figure 2.**
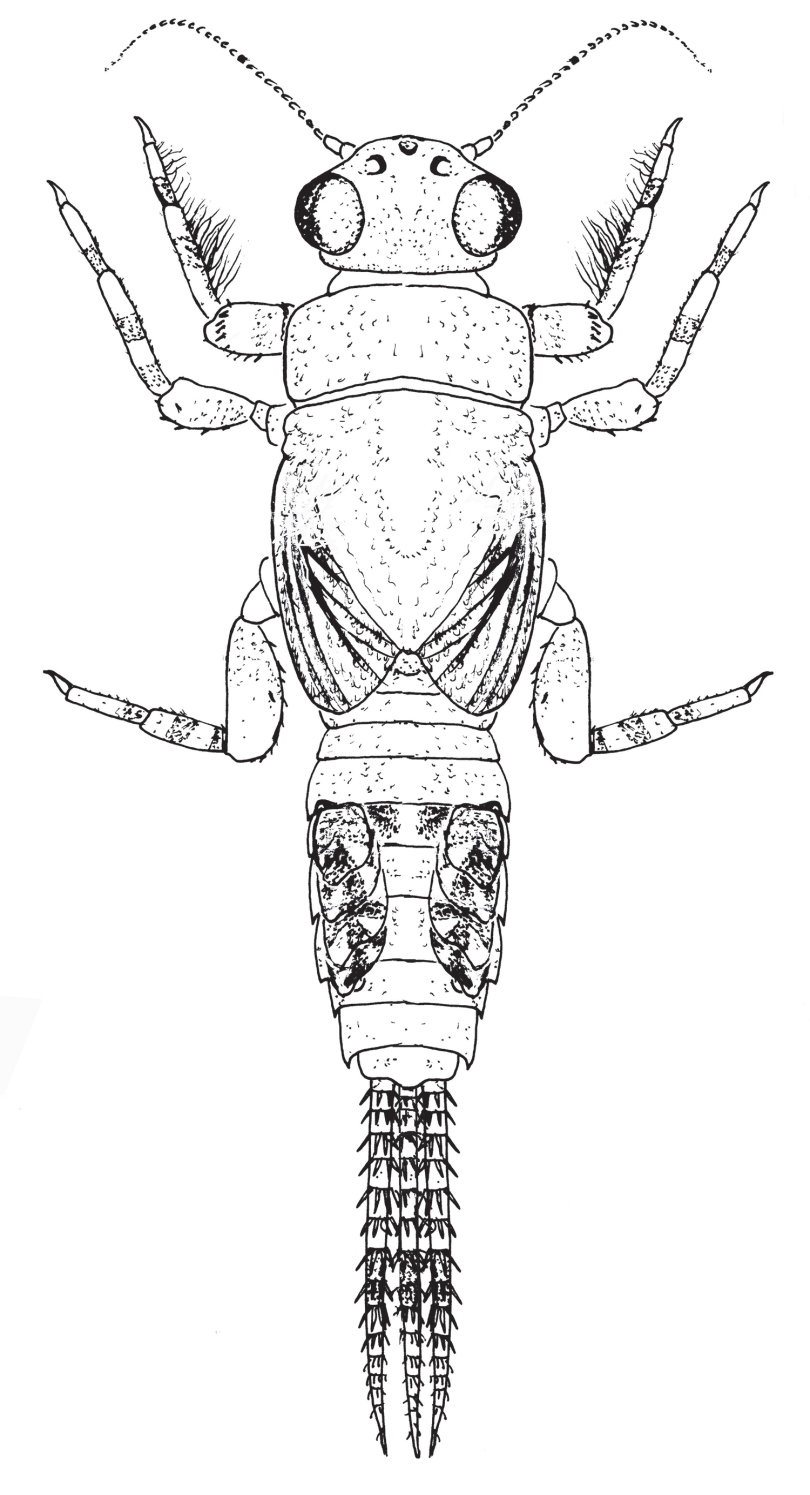
Male nymphal habitus of *Teloganopsis setosa* sp. n.

**Figure 3.**
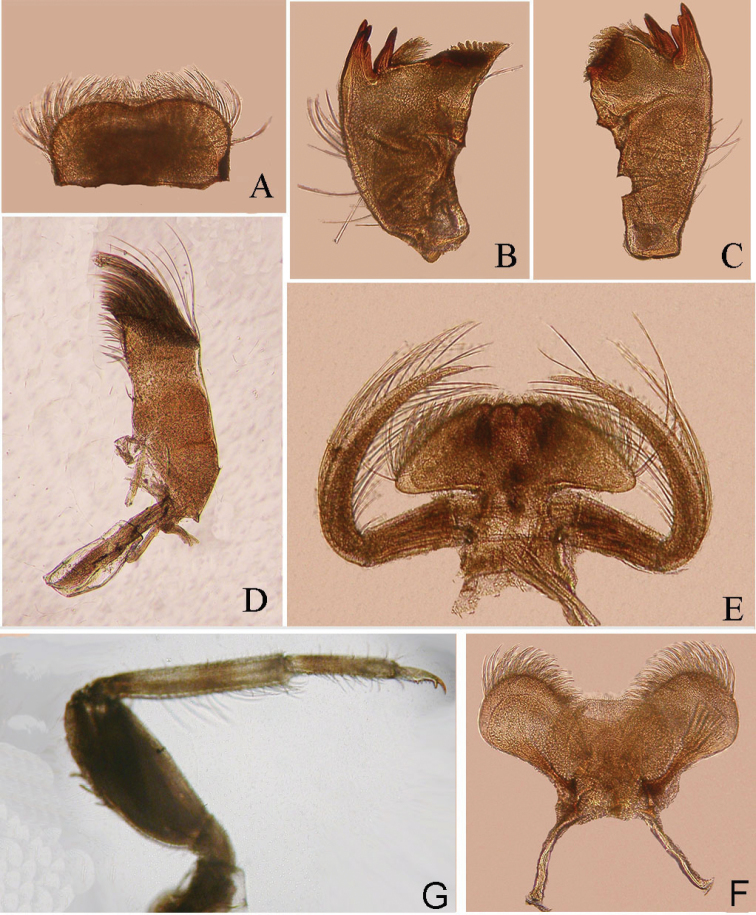
Structures of *Teloganopsis setosa* sp. n. nymph (digital photographs) **A** labrum (dorsal view) **B** right mandible **C** left mandible **D** maxilla **E** labium (ventral view) **F** hypopharynx (dorsal view) **G** foreleg.

**Figure 4.**
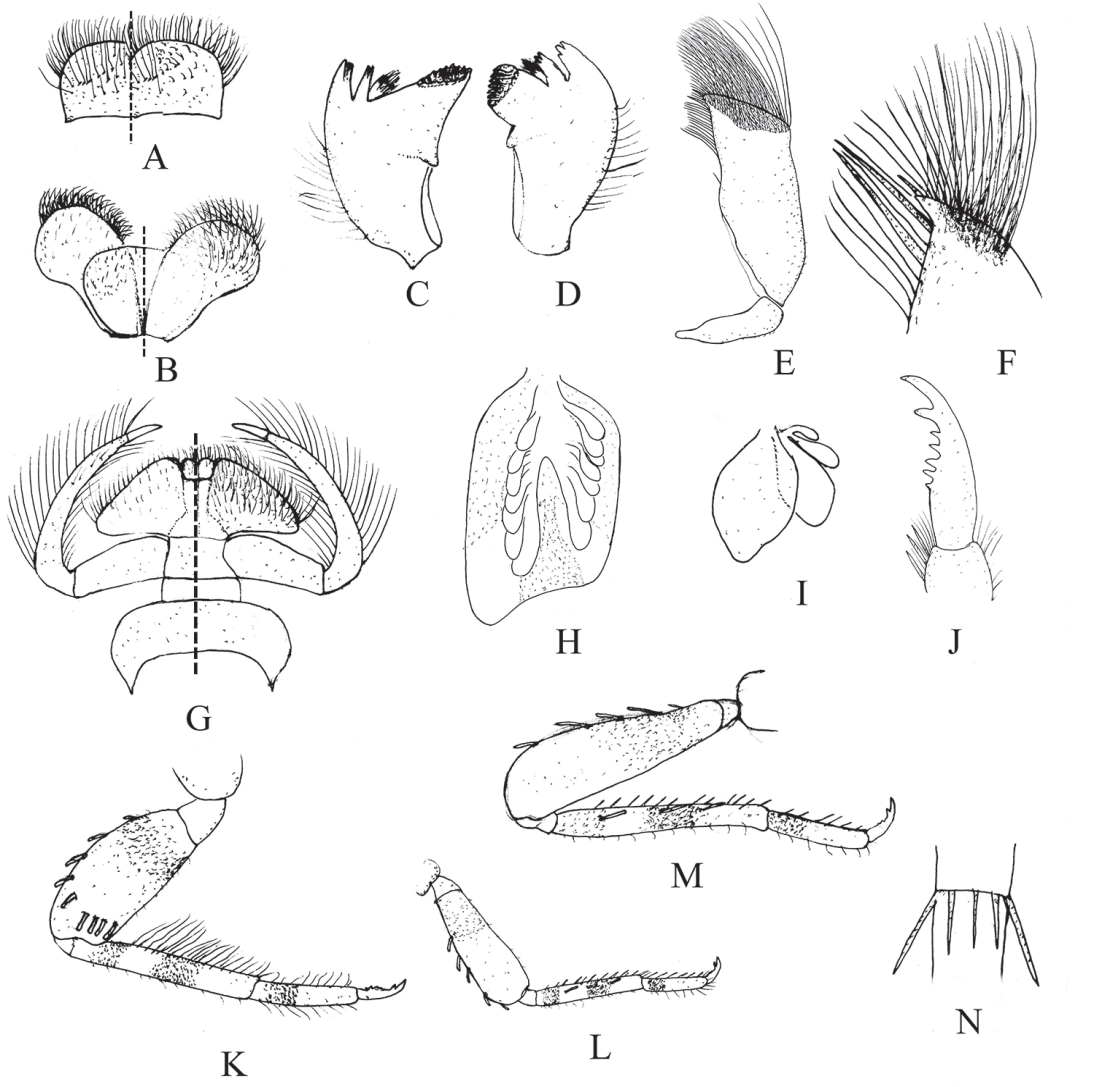
Nymphal characters of *Teloganopsis setosa* sp. n. **A** labrum (dorsal view on left; ventral view on right) **B** hypopharynx (dorsal view on left; ventral view on right) **C** right mandible **D** left mandible **E** maxilla **F** apex of maxilla **G** labium (dorsal view on left; ventral view on right) **H** gill I (ventral view) **I** gill V (dorsal view) **J** claw **K** foreleg **L** midleg **M** hindleg **N** articulation of caudal filaments.

**Figure 5.**
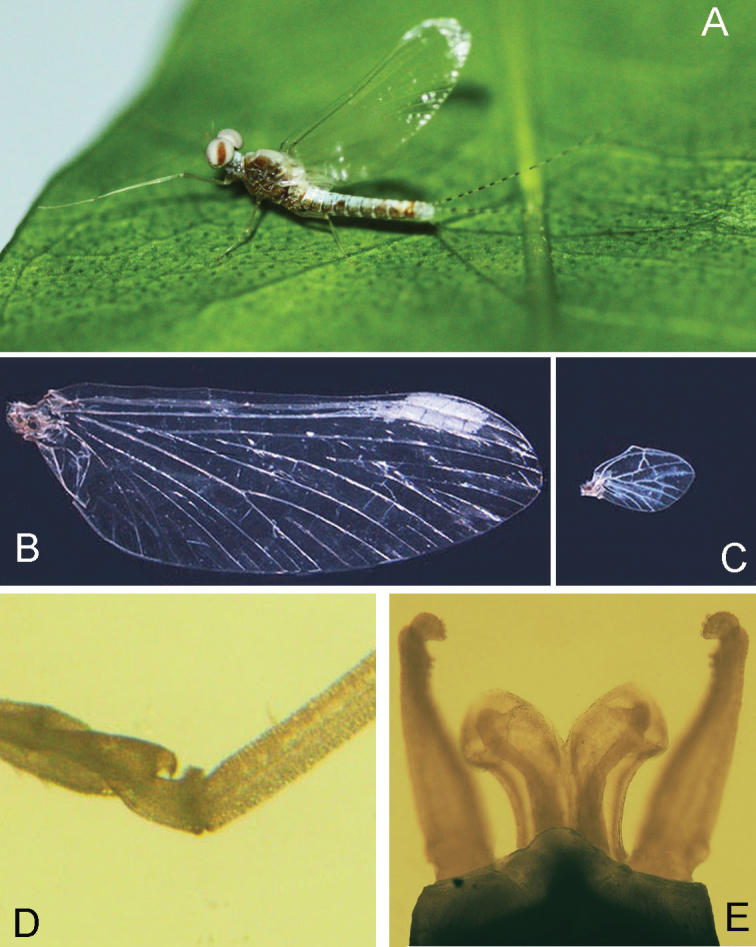
Male structures of *Teloganopsis setosa* sp. n. (digital photographs) **A** male adult **B** forewing **C** hindwing **D** proximal hook of foreleg **E** genitalia (ventral view).

**Figure 6.**
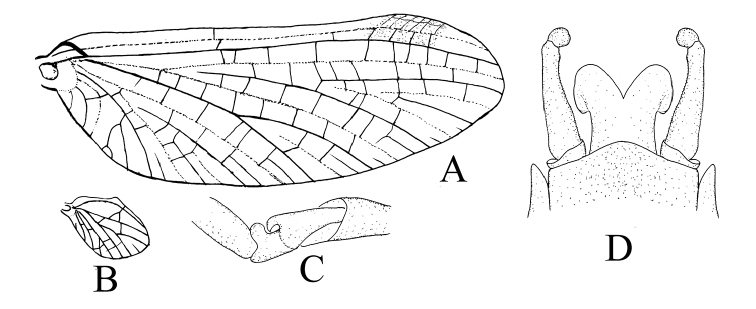
Male structures of *Teloganopsis setosa* sp. n. **A** forewing **B** hindwing **C** joint of tarsi and tibiae of foreleg **D** genitalia (ventral view).

**Figure 7.**
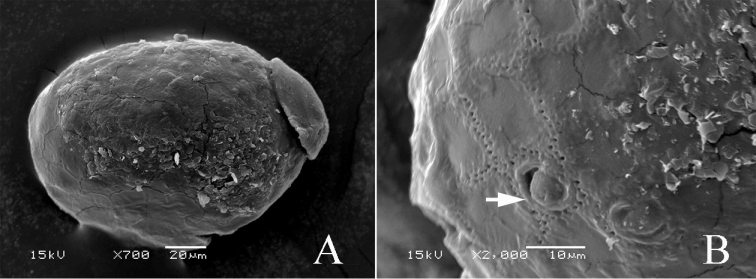
*Teloganopsis setosa* sp. n. Egg morphology (SEM photos) **A** whole egg **B** egg surface enlarged (arrow indicates the projection).

Figs 1 , 2 , 3 , 4 , 5 , 6 , 7

#### Holotype.

♂, Ba-Wang-Ling National Forest Park (19°12.12′N, 109°09.35′E, alt. 300 m), Chang-Jiang county, Hainan Province, China, collected by Qin SI, Jun-Zhi SUN, Juan-Yan LUO, 16 Nov 2015; **Paratypes**: 2♂♂1♂subimago 2♀♀30L, same as the holotype.

#### Diagnosis.

**Nymph**: Inner margins of forelegs and mouthparts (labrum, mandibles, and labium) have dense and long setae. Paraglossae and palpi of labium are enlarged. Abdominal tergum IV has two dark spots. **Male adults**: The dorsolateral projections of the penes are visible in ventral view. The caudal filaments have wide reddish-brown bands. Two pigmented spots or dots are on tergum IV.

#### Description.

***Nymph*** (in alcohol, Figs 1–4): *Body length* 3.0–5.0 mm, caudal filaments 1.2–1.6 mm, cerci subequal to terminal filament (Figs 1, 2). Body reddish to dark brown. Head hypognathous, antennae located near front margin of head, with very tiny setae on articulations of flagellae; antennal length approximately 1.5X head width. Dark base of ocelli and male compound eyes clear.

*Mouthparts*: *labrum* with relatively long setae on dorsal surface and free margin, ventral surface with shorter but stouter setae (Figs 3A, 4A). Both *mandibles* with long setae on outer surface; outer and inner incisors of left mandible divided into three teeth apically, but inner incisor of right mandible serrated into two teeth only; prosthecae of mandibles with a tuft of spines on common base (Figs 3B–C, 4C–D). *Maxillae*: dense long setae and bristles on apex and dorsal surface (Fig. 4F); two rows of bristles on apical half of inner margin; basal half of outer margin and cardo also with shorter setae (Figs 3D, 4E). *Labium*: paraglossae enlarged, its posterolateral angle projected significantly, this makes paraglossae triangular; ventral surface of glossae with denser and longer setae than dorsal surface; labial palpi elongated remarkably into long broad filamentous process-like structure, 3-segmented, basal and apical one smooth; basal one slightly longer than half of segment II; apical one very short, less than half of basal one; segment II with very long and distinct setae on lateral margins and dorsal surface (Figs 3E, 4G); *hypopharynx* with denser and longer setae on superlinguae surface, other parts of lingua and superlinguae with shorter setae (Figs 3F, 4B).

*Legs* (Figs 3G, 4K–M): femora of all legs slightly shorter than tibiae, tarsi slightly longer than half tibiae; basal half of femora, tibiae and tarsi darker or more reddish brown than other parts, an additional reddish to dark ring on median tibiae; foreleg with blunt but relatively long bristles on outer margin of femora, 4–5 more bristles lined up a row sub-apically on femoral surface; inner surface of fore-tibiae and tarsi with rows of setae, those setae of tibiae longer than others; outer margin of them with shorter setae (Figs 1, 2, 3G, 4K); mid- and hind legs similar in structure: outer margin of femora with 3–5 bristles, tibiae with 2–4 bristles on dorsal surface, rows of spines on inner margin while outer margin with tiny setae; setae and spine pattern of tarsi similar to tibiae but without bristle; inner margin of all femora with tiny setae too (Fig. 4L, M); *claws* of all legs similar, with six denticles from middle to apex, apical one larger than others (Figs 3G, 4J).

*Abdomen*: reddish brown to brown, tergum IV with a pair of clear reddish to dark dots, terga V–VI distinctly paler than others; terga washed with brown to dark pigments without regular markings (Figs 1, 2). Sterna generally brown to dark brown but with pale median line, especially on posterior half; an additional pair of oblique dark stripes present laterally. *Gills* on terga III–VII, anterior four pairs of gills similar in structure: dorsal lamellae plate-like, with tri-lobed marking dorsally; ventral lamellae bifurcated into two parts, each one with 4–6 leaf-like lobes (Fig. 4H); gills on tergum VII much smaller than anterior ones, ventral lamellae divided into three lobes (Fig. 4I). All posterolateral corners of terga IV–IX extended into small but sharp angles, progressively larger posteriorly (Figs 1, 2). Posterior and lateral margins of each tergum with tiny spines. Caudal filaments with reddish to dark median band (Figs 1, 2); articulations with distinct spines (Fig. 4N).

***Male imago*** (in alcohol, Figs 5–6). *Body length* 5.0–6.0 mm, caudal filaments 7.0–8.0 mm, forewing 6.5–7.0 mm, hindwing only approx. 1/5 of forewing in length. Upper portion of compound eyes reddish, basal portion dark, two eyes separated with a distance less than width of median ocellus (Fig. 5A). *Forewings* hyaline, but subcostal brace and bulla of Sc pigmented with reddish brown dots; cross veins in stigma area oblique, those between C and Sc separated into two parts by a short vein (Figs 5B, 6A). *Hindwings* transparent, with a projection at median leading margin (Figs 5C, 6B). Femora subequal to tarsi in length of foreleg, each of them approx. 2/3 of tibiae. Tarsi 5-segmented, basal one the shortest, with hook-like structure near tibiae (Figs 5D, 6C); other segments of fore-tarsi progressively shorter apically. Mid- and hind-leg similar: femora distinctly shorter than tibiae, tarsi less than half of tibiae; tarsi 4-segmented, length arrangement in decreasing order as 4, 1, 2, 3. All *claws* of legs similar, one blunt and one hooked. *Abdominal terga* pale to brown, tergum IV with a pair of reddish to dark dots dorsally (Fig. 5A). *Sterna* pale but with reddishly pigmented lateral margins. Basal half of each segment of caudal filaments reddish, apical half pale (Fig. 5A).

*Genitalia* (Figs 5E, 6D): basal segment of forceps very short, less than 1/6 of segment II; the latter straight, narrowed progressively from base to apex, with tiny projections on inner margin; apical segment approx. 1/6 to 1/5 of segment II, mesal margin emarginated at base, making segment III appear somewhat pointed or hooked; penes fused at 2/3 base and separated at apical 1/3; each penis with broad lobe-like projection dorsally; posterior margin of subgenital plate convex.

***Female imago*** (in alcohol). *Body length* 6.5–7.0 mm, forewing 7.5–8.0 mm, caudal filaments 6.5–7.0 mm. Body pale to pale reddish, washed with reddish brown dots and markings laterally. *Forewings* transparent but subcostal brace and bulla of Sc with dark pigments; *Tergum* IV also with a pair of brown dots but much smaller and indistinct than those of male. *Sterna* dark brown with pairs of pale dots. Tibiae longer than femora, the latter much longer than tarsi. One of *claws* blunt, the other hooked and sharp.

***Male Subimago*** (in alcohol). *Body length* 5.0 mm, forewing 6.5–7.0 mm, caudal filaments 5.0 mm; resembles male imago except dull; veins and cross veins of wings much clearer than male imago.

***Egg*** (Fig. 7). Egg scanned with length 0.133 mm, width 0.084 mm. Egg oval with one polar helmet-shaped cap (Fig. 7A). Egg surface sculptured with hexagonal structures and decorated with sparse tubercle-like projections (Fig. 7B).

#### Distribution.

China (Hainan Province).

#### Etymology.

The name *setosa* (from Latin epithet *setosus*) means having numerous setae. It indicates here that the nymphs of the new species have long and dense setae on the mouthparts and forelegs.

#### Remarks

Based on the following characters (gills on abdominal segments III–VII, ventral lamellae of gills IV (on segment VI) bifurcated, body without any tubercles or projections, maxillae without palpi and shape of claw), the nymph of this new species belongs to the genus *Teloganopsis* based on the nymphal keys provided by Jacobus and McCafferty (2008) and Ubero-Pascal and Sartori (2009). The proximal hook of the forelegs, the shape of the penis, and forceps also imply that the male imagoes of this new species belong to the genus *Teloganopsis*.

In the nymphal key of all known species of the genus *Teloganopsis* prepared by Ubero-Pascal and Sartori (2009), *T. setosa* sp. n. is most similar to the species *T. puigae* because of their uniform body color pattern, missing maxillary palpi, and without any tubercles on body. However, the setal pattern on the forelegs and the shapes of maxillae and labia are different. The nymphs of *T. setosa* sp. n. have longer setae on forelegs and longer labial palpi. In the imaginal stage of these two species, the shape of their hindwings and abdominal colour patterns are dissimilar. The hindwing of *T. setosa* sp. n. has a shallow marginal projection while the counterpart in *T. puigae* has a larger and sharper projection. Furthermore, the imaginal abdomen of the latter species is uniformly dark while that of *T. setosa* sp. n. is pale with a pair of reddish brown dots on tergum IV.

Among Chinese ephemerelliids belonging to the genus *Teloganopsis*, the nymphs of *T. setosa* sp. n. is closest to *T. punctisetae* (Matsumura, 1931) and *T. jinghongensis* (Xu et al., 1984) as they have no maxillary palpi nor any tubercles on the body. As imagoes, they are also similar because of their longer tibiae (distinctly longer than the femora and tarsi) on the forelegs and the morphology of the genitalia (penis with dorsal projection).

**Figure 8.**
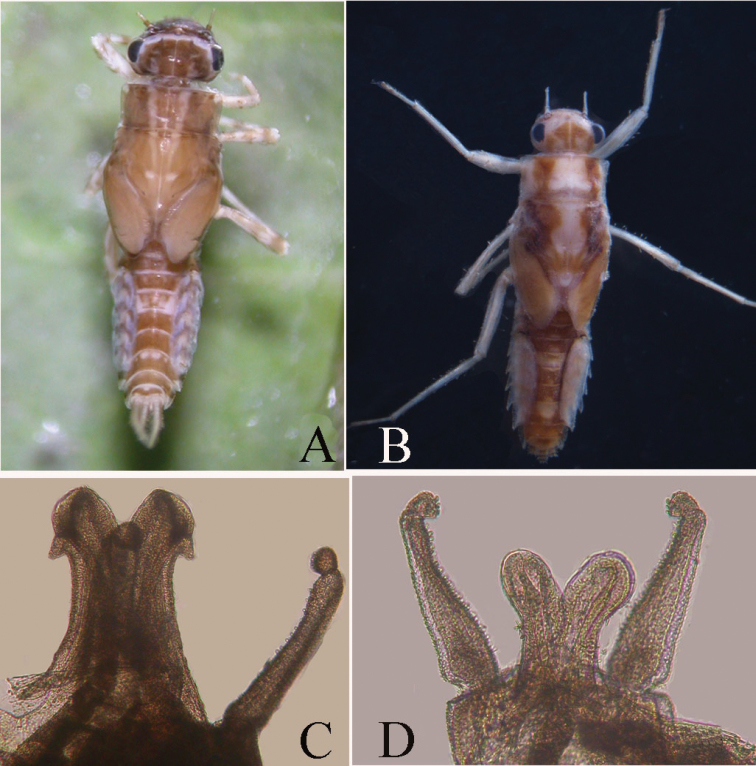
Structures of *Teloganopsis punctisetae* and *T. jinghongensis* (digital photographs) **A** *T. punctisetae* nymph (dorsal view) **B** *T. jinghongensis* nymph (dorsal view) **C** *T. punctisetae* genitalia (ventral view) **D** *T. jinghongensis* genitalia (ventral view).

However, the nymph of *T. setosa* sp. n. is unique because of its setaceous mouthparts and forelegs. Four diagnostic characters are remarkable: 1) the outer margin of mandible has long setae (Figs 3B–C, 4C–D); 2) the paraglossae of the labium are expanded and bear dense setae ventrally (Figs 3E, 4G); 3) segment II of the labial palpi are greatly elongated and have setae dorsally (Figs 3E, 4G); 4) forelegs possess long setae (Figs 1, 2, 3G, 4K). The absence of projections, spines, or tubercles on nymphal body also contributes to its identification (Figs 1, 2). Besides these characters, when compared to the similar species *T. punctisetae* and *T. jinghongensis*, the nymph of this new species has more and longer setae on its mouthparts (the setae on the labrum, maxillae, hypopharynx, and labium are more numerous and denser). The nymphal femora of the new species are broader, with fewer spines compared to those of *T. punctisetae* and *T. jinghongensis*, and their color pattern is different. Tergum IV of *Teloganopsis setosa* sp. n. is more colorful than that of these two species (Fig. 1), while the latter two species have longitudinal pale stripes on body (Fig. 8A, B), which are not present in the new species.

In male imagoes, however, the distinguishing characters of the new species mostly rely on color; 1) the penes and dorsolateral projections of the penes are larger and more distinctive than those of *T. punctisetae* and *T. jinghongensis* (Figs 5E, 6D, 8C, 8D); 2) the caudal filaments of *T. setosa* sp. n. have broad reddish brown bands on the basal half of each segment (Fig. 5A) while those of the *T. punctisetae* and *T. jinghongensis* have rings on the articulations, while broad bands are rare; 3) both the male and female of *T. setosa* sp. n. have relatively obvious and big spots on tergum IV (Fig. 5A) but in *T. punctisetae* and *T. jinghongensis* there are no recognizable markings on the terga.

#### Key to the three close Chinese *Teloganopsis* species (adult)

**Table:** 

1	Projection of penis broad, visible in ventral view (Figs 5E, 8C)	**2**
–	Projection of penis smaller, invisible in ventral view (Fig. 8D)	***Teloganopsis jinghongensis***
2	Tergum IV with clear reddish spots (Fig. 5A); caudal filaments with reddish brown bands on basal half segment (Fig. 5A)	***Teloganopsis setosa* sp. n.**
–	Tergum without spots; only articulations of caudal filaments with rings	***Teloganopsis punctisetae***

#### Key to the three close Chinese *Teloganopsis* species (nymph)

**Table:** 

1	Inner margin of forelegs with long setae (Figs 1, 2, 3G, 4K); paraglossae of labium and labial palpi greatly enlarged (Figs 3E, 4G)	***Teloganopsis setosa* sp. n.**
–	Forelegs without long setae; paraglossae of labium and its palpi not enlarged	**2**
2	Head to abdominal segment III with three longitudinal pale stripes (Fig. 8A); maxillae brush-like	***Teloganopsis punctisetae***
–	One broad pale line on dorsal body (Fig. 8B); maxillae with less setae but more spines dorsally	***Teloganopsis jinghongensis***

## Discussion

Mayflies with setaceous mouthparts, elongated maxillary labial palpi, and long setae on the forelegs have been found in several lineages in the order Ephemeroptera, such as *Isonychia* (Isonychiidae), *Nathanella* (Leptophlebiidae), *Rhoenanthus* (Potamanthidae), *Oligoneuriella* (Oligoneuriidae), *Tricorythus* (Tricorythidae), and *Clypeocaenis* (Caenidae). In the Ephemerellidae, as far as we know, no similar form has been reported. This new species represents a new evolutionary type in the family, but compared to related species such as *T. punctisetae* and *T. jinghongensis*, their nymphal body patterns are similar, and the adults are alike. Most importantly, the setaceous mouthparts and brush-like maxillae are also found in *T. punctisetae* at least, so there is no need to erect a new generic level for it. Based on the habit, habitat, and behavioural information provided by Needham et al. (1935), Edmunds et al. (1976), and Elpers and Tomka (1995), the mayfly nymphs with setaceous mouthparts and forelegs are usually filter-feeding. Based upon its characters, the new species described in this paper is also believed to be filter-feeder. Most ephemerellid nymphs usually live in and feed on the aquatic spirogyra or branches and leaves stacked in the lentic or lotic water. The *Teloganopsis setosa* sp. n. nymph may have evolved the ability to collect organic particles from flowing water using their long and dense setae on forelegs and mouthparts, just like the *Isonychia* and *Clypeocaenis* mayflies.

## Supplementary Material

XML Treatment for Teloganopsis setosa
